# Incidence of Metastasis in the Central Nervous System in Advanced Breast Cancer Treated With CDK 4/6 Inhibitors: A Multicenter, Retrospective Study

**DOI:** 10.1002/mco2.70221

**Published:** 2025-05-24

**Authors:** Yan‐Ling Wen, Xi‐Wen Bi, Xue‐Wen Zhang, Si‐Fen Wang, Chang Jiang, Li Wang, Yong‐Yi Zhong, Yuan‐Yuan Huang, Jian‐Li Zhao, Qian‐Jun Chen, Cong Xue, Zhong‐Yu Yuan

**Affiliations:** ^1^ Department of Medical Oncology State Key Laboratory of Oncology in South China Collaborative Innovation Center for Cancer Medicine Guangdong Provincial Clinical Research Center for Cancer Sun Yat‐sen University Cancer Center Guangzhou China; ^2^ Department of Anesthesiology State Key Laboratory of Oncology in South China Collaborative Innovation Center for Cancer Medicine Guangdong Provincial Clinical Research Center for Cancer Sun Yat‐sen University Cancer Center Guangzhou China; ^3^ Breast Tumor Center Sun Yat‐Sen Memorial Hospital Sun Yat‐Sen University Guangzhou China; ^4^ Department of Breast Oncology Traditional Chinese Medicine Hospital of Guangdong Province Guangzhou Guangdong China

**Keywords:** advanced breast cancer, CDK 4/6 inhibitor, CNS metastasis, endocrine therapy

## Abstract

Central nervous system (CNS) metastasis remains a major cause of mortality in advanced breast cancer (ABC). While cyclin‐dependent kinase 4/6 inhibitors (CDKIs) combined with endocrine therapy (ET) delay resistance in hormone receptor (HR)‐positive and human epidermal growth factor receptor 2 (HER2)‐negative ABC, their impact on CNS metastasis development has not been fully elucidated. This retrospective study analyzed 435 ABC patients without baseline CNS metastases who received first‐line ET with or without CDKIs across three Chinese hospitals (August 2018–July 2022). Primary end points included CNS as the first metastatic site, CNS metastasis‐free survival (CNSM‐FS), and CNS metastasis incidence over time. Secondary end points were progression‐free survival (PFS) and overall survival (OS). The results indicated that the addition of CDKIs to ET significantly reduced the incidence of CNS as the first site of metastasis (3.7% vs. 9.5% with ET alone; *p* = 0.0015) and extended CNSM‐FS (71.6 months vs. 63.6 months, respectively; hazard ratio [HR], 0.53; 95% CI, 0.31–0.92). Overall, CNS metastasis incidence was lower with ET + CDKIs (7.9% vs. 15.5%, *p* = 0.014), and improvements were observed in both PFS and OS. These findings suggest that ET + CDKIs as first‐line therapy in ABC may reduce CNS metastasis risk and extend CNSM‐FS, offering a potential strategy for preventing CNS metastases.

## Graphical abstract

Among 449 advanced HR+/HER2− breast cancer patients screened (August 2018–July 2022), 14 were excluded for CNS relapse. The remaining patients (*n* = 435) were allocated to either ET + CDKI (*n* = 215) or ET alone (*n* = 220), with subsequent comparative analysis of CNS metastasis incidence, CNSM‐FS, PFS, and OS.

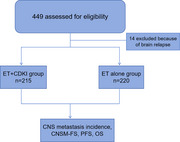

## 1 Introduction

Approximately 70% of patients with advanced breast cancer (ABC) have hormone receptor (HR)‐positive and human epidermal growth factor receptor 2 (HER2)‐negative (HR+/HER2−) disease and are commonly treated with endocrine‐based therapies [1, 2, 3]. The US Food and Drug Administration (FDA) has granted approval for the use of cyclin‐dependent kinase 4/6 inhibitors (CDKIs) in conjunction with endocrine therapy (ET) for both first‐line and second‐line treatment of HR+/HER2− ABC. This combination treatment demonstrates clinically significant outcomes, including a higher objective response rate, extended progression‐free survival (PFS), and improved overall survival (OS) compared to ET alone [4, 5, 6, 7].

The central nervous system (CNS) is a frequent site for metastasis in breast cancer (BC) [8, 9], portending poor survival owing to its rapid development and a lack of efficient treatment methods for intracranial lesions [10, 11]. Although the incidence of CNS metastases in HR+/HER2− ABC is much lower than that in either HER2‐positive or triple‐negative ABC, it still ranges from 8.3% to 15%, with a median OS of 14 (4–34) months [12, 13, 14, 15, 16, 17]. ET can improve treatment outcomes for patients with HR+/HER2− ABC. However, CNS metastasis is a major clinical issue that increases the morbidity and mortality of patients with HR+/HER2− ABC [17]. Therefore, CNS metastasis in ABC is attracting increasing research interest.

Multiple preclinical and clinical studies have suggested that CDKIs can penetrate the blood‐brain barrier (BBB) and exhibit CNS activity [18, 19, 20, 21, 22]. Abemaciclib demonstrated the ability to penetrate the BBB due to its substrate for P‐glycoprotein (P‐gp) and BC resistance protein (BCRP), which are two efflux transporters on the BBB, and significantly extended survival in an orthotopic U87MG intracranial glioblastoma xenograft model [20, 23]. A Phase I clinical research on malignant brain tumors showed that potential therapeutic ribociclib concentrations could be achieved in both CSF and tumor tissue; the mean tumor‐to‐plasma ratio of ribociclib was 19.8 (range: 2.22–53.4) [24]. Hence, they can provide intracranial clinical benefits in HR+/HER2− ABC with CNS metastases [22, 25]. On the other hand, CDKs and cyclin D1 (CCND1)—a key downstream target of estrogen receptor (ER) signaling—play a pivotal role in cell‐cycle progression. In HR+ BC, CDK4/6‐CCND1 axis hyperactivation drives endocrine resistance, making it a critical therapeutic target [26, 27, 28]. Also, emerging evidence suggests that CDK pathway alterations may also facilitate CNS metastasis, as genomic analyses identify CDK dysregulation in brain metastases (BM) across tumor types [29, 30]. Consequently, this protective effect may be attributed to their dual mechanisms of action: direct penetration of the blood‐brain barrier to target micrometastases and systemic control of extracranial disease that potentially prevents CNS dissemination. However, limited information is available on whether adding CDKIs to ET affects the occurrence of CNS metastasis in ABC patients.

Given the compelling preclinical and clinical evidence, it can be reasonably postulated that CDKIs can reduce CNS metastases in BC. To further validate this assumption, we conducted this retrospective analysis to evaluate the impact of CDKIs on the incidence of CNS metastasis in ABC patients. This study aims to gather new data regarding the risk of CNS metastasis in patients with ABC receiving ET ± CDKIs as a first‐line therapy and provide novel insights into CNS‐directed preventive strategies for ABC patients.

## 2 Results

### 2.1 Baseline Characteristics

From August 2018 to July 2022, 449 patients with advanced HR+/HER2− BC were screened across three study centers. Of these, 14 patients were excluded because of CNS metastases at the time of recurrence, leaving 435 eligible patients who were ultimately included in the study. Of these, 215 (49.4%) were included in the ET + CDKI group and 220 (50.6%) in the ET alone group. The baseline characteristics of the patients were evenly distributed between the two groups (Table 1). The median age was 49 years (IQR: 42–56). A total of 56 patients (12.9%) had de novo ABC (12.6% in the ET + CDKI group and 13.2% in the ET alone group). The DFI at baseline was more than 24 months in 253 patients (58.2%). Visceral metastases, such as the liver, lungs, and other internal sites, were identified in 198 patients (45.5%), while bone‐only disease was present in 86 (19.8%). Among the 435 patients, 118 (27.1%) had received aromatase inhibitors, and 196 (45.1%) received tamoxifen as adjuvant ET; the others had not received adjuvant ET.

**TABLE 1:** Characteristics of the Patients at Baseline.

Category	ET + CDKI (*N* = 215)	ETAlone (*N* = 220)
Age, median years (range)	49.6 (41.1–56.6)	48.4 (42.2–55.6)
≤ 55	152 (70.7)	165 (75.0)
> 55	63 (29.3)	55 (25.0)
Estrogen receptor status		
Positive	210 (97.7)	205 (93.2)
Negative	1 (0.5)	12 (5.5)
Other or not reported	4 (1.9)	3 (1.4)
Progesterone receptor status		
Positive	177 (82.3)	193 (87.7)
Negative	33 (15.3)	23 (10.5)
Other or not reported	5 (2.3)	4 (1.8)
Menopausal status^a^		
Premenopausal	89 (41.4)	157 (71.4)
Postmenopausal	126 (58.6)	63 (28.6)
Prior treatment		
Chemotherapy	164 (76.3)	164 (74.5)
Neoadjuvant chemotherapy	29 (13.5)	18 (8.2)
Adjuvant chemotherapy	146 (67.9)	154 (70.0)
Adjuvant endocrine therapy	156 (72.6)	158 (71.8)
Tamoxifen	72 (33.5)	124 (56.4)
Aromatase inhibitors	84 (39.1)	34 (15.5)
Disease‐free interval (DFI)^b^		
De novo ABC	27 (12.6)	29 (13.2)
Existing disease		
≤ 24 month	66 (30.7)	50 (22.7)
> 24 month	113 (52.6)	140 (63.6)
Ki‐67 index, %		
< 20	100 (46.5)	89 (40.5)
≥ 20	114 (53.0)	52 (23.6)
Other or not reported	1 (0.5)	79 (35.9)
Site of metastases		
Bone	118 (54.9)	111 (50.5)
Only	39 (18.1)	47 (21.4)
Any	79 (36.7)	64 (29.1)
Visceral	94 (43.7)	104 (47.3)
Lung	62 (28.8)	66 (30.0)
Liver	38 (17.7)	46 (20.9)
Lymph nodes	95 (44.2)	102 (46.4)
Breast	19 (8.8)	12 (5.5)
First on‐study ET		
Aromatase inhibitors	122 (56.7)	160 (72.7)
Fulvestrant	92 (42.8)	36 (16.4)
Tamoxifen/ Toremifene	1 (0.5)	18 (8.2)

*Note*: Data are presented as no. (%) unless otherwise noted. Where values do not add up to 100%, the remaining data are missing, unavailable, or could not be assessed. Data for DFI and previous endocrine therapy were available only for patients who were diagnosed initially with early breast cancer and then experienced a relapse of the disease.

Abbreviations: CDK4/6i, cyclin‐dependent kinase 4/6 inhibitor; ET, endocrine therapy.

^a^ Menopausal status is at the time of diagnosis.

^b^ DFI was defined as the time from the diagnosis of primary breast cancer to the first recurrence in patients who received (neo)adjuvant therapy.

### 2.2 Efficacy

**FIGURE 1:**
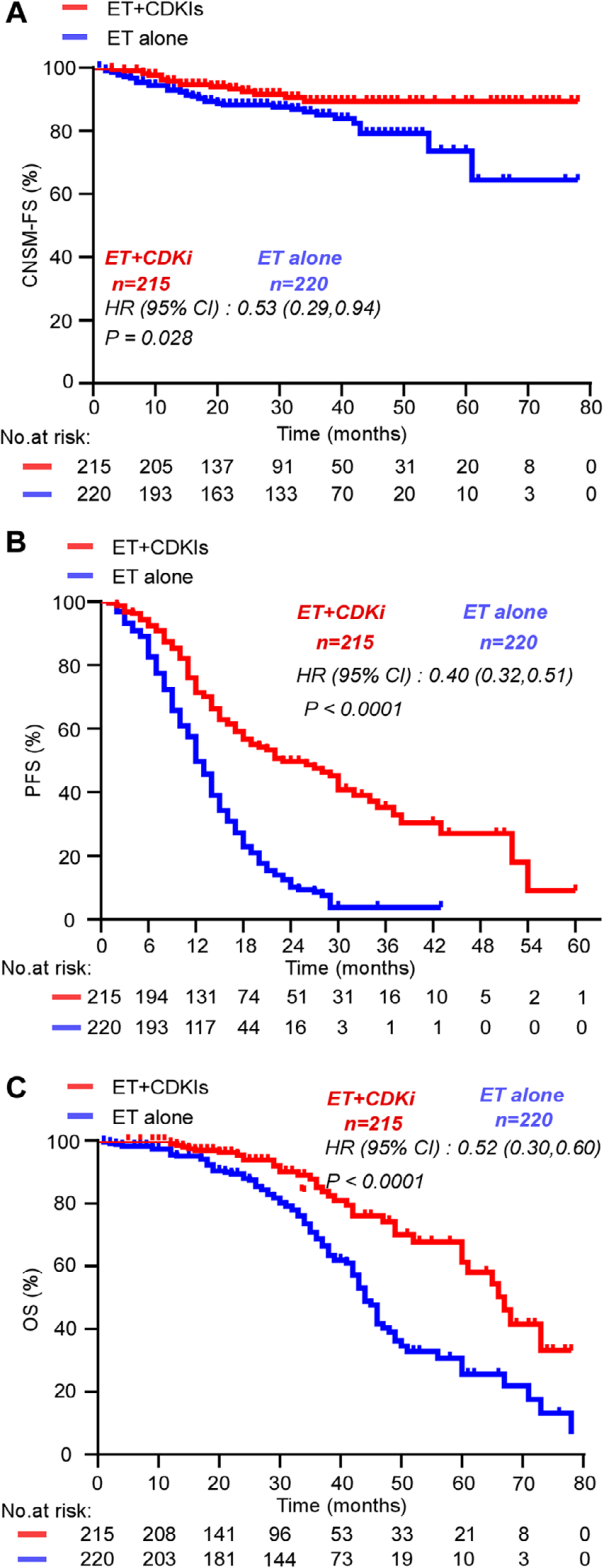
Kaplan–Meier curve for (A) CNS metastasis‐free survival (CNSM‐FS); (B) progression‐free survival (PFS); (C) overall survival (OS).

CNS metastases occurred in 11.7% of the abovementioned 435 patients, with a median follow‐up time of 35 (32.8–37.2) months. CNS as the first metastasis site was recorded in 3.7% of patients (8 of 215 patients) in the ET + CDKI group and in 9.5% (21 of 220 patients) in the ET alone group. This difference in the incidence rate was statistically significant (treatment differences, 5.8%; 95% CI, 1.2–10.5; *p* = 0.015) (Table 2). CNSM‐FS was longer for the ET + CDKI group than it was for the ET alone group (71.6 months [95% CI, 68.7–74.6] vs. 63.6 months [95% CI, 58.6–68.7], respectively; HR, 0.53; 95% CI, 0.31–0.92) (Figure 1A). The overall incidence of CNS metastasis over time was also lower in the ET + CDKI group [17 of 215 patients (7.9%)] than it was in the ET alone group [34 of 220 patients (15.5%); *p* = 0.014], with a median time of 12 (8.6–15.4) and 15 (9.3–20.7) months for the first CNS metastasis to occur in the ET + CDKI and ET alone groups, respectively (Table 2).

**TABLE 2:** Efficacy results of CNS.

CNS end points	ET + CDKI	ET alone	OR	95% CI	*p* ^a^
No. of patients	215	220			
CNS as the first metastasis site			0.68	0.53–0.87	0.015
No. of patients (%)	8 (3.7)	21 (9.5)			
95% CI, %	1.2–6.3	5.6–13.5			
Incidence of CNS metastasis over time			0.51	0.30–0.89	0.014
No. of patients (%)	17 (7.9)	34 (15.5)			
95% CI (%)	1.2–6.3	5.6–13.5			
Time to first CNS metastasis, months					
Median	12	15			
Range	8.6–15.4	9.3–20.7			

Abbreviations: CDKI, cyclin‐dependent kinase 4/6 inhibitor; CI, confidence interval; CNS, central nervous system; ET, endocrine therapy; OR, odds ratio.

^a^ The *p* values for the incidence of CNS as the first metastasis site and CNS metastasis over time were computed using chi‐square tests.

In a multivariate analysis that adjusted for covariates with moderate correlation, any variables linked to CNS metastases (*p* < 0.2) were included; ET + CDKIs was independently correlated with lower incidence of CNS as the first metastasis site (OR 2.70; 95% CI, 1.10–6.58, *p* = 0.029) and incidence of CNS metastasis over time (OR 2.16; 95% CI, 1.16–4.01, *p* = 0.015; Tables 3 and 4).

**TABLE 3:** Univariate and multivariate analysis of variables that influence CNS as the first metastasis site.

	Univariate		Multivariate	
Variable	OR (95% CI)	*p* value	OR (95% CI)	*p* value
Treatment		**0.019**		
ET + CDKI	1		1	
ET alone	2.73 (1.18–6.31)		2.70 (1.10–6.58)	0.029*
Age		0.708		
≤ 55	1			
> 55	0.85 (0.35–2.04)			
Menopausal status		0.877		
Premenopausal	1			
Postmenopausal	1.32 (0.50–2.27)			
Previous chemotherapy		0.350		
Yes	1			
No	1.61 (0.60–4.33)			
Previous ET				
No	1		1	
Tamoxifen	2.11 (0.76–5.88)	**0.154**	1.99 (0.69–5.68)	0.204
Aromatase inhibitors	1.40 (0.43–4.54)	0.575	1.86 (0.55–6.32)	0.319
Disease‐free interval				
Newly diagnosed disease	1			
Existing disease				
≤ 24 month	1.14 (0.28–4.56)	0.859		
> 24 month	1.43 (0.41–5.03)	0.573		
Lung or liver involvement				
No	1		1	
Liver	1.14 (0.36–3.64)	0.830	1.09 (0.33–3.53)	0.881
Lung	1.80 (0.76–4.31)	**0.185**	1.96 (0.81–4.77)	0.136
Both	5.11 (1.26–20.79)	**0.023**	4.49 (1.07–18.80)	0.404
Bone involvement				
No	1			
Any	0.81 (0.33–1.99)	0.650		
Only	1.22 (0.47–3.12)	0.686		
First on‐study ET		0.501		
Aromatase inhibitors	1			
Fulvestrant	1.32 (0.59–2.97)			

*Note*: OR represents the odd ratio for the incidence of CNS as the first metastasis site.

Abbreviations: CDKI, cyclin‐dependent kinase 4/6 inhibitor; CI, confidence interval; CNS, central nervous system; ET, endocrine therapy; OR, odd ratio.

Bold values indicate *p* < 0.2, which were included in the subsequent multivariate analysis.

* *p* <0.05.

**TABLE 4:** Univariate and multivariate analysis of variables that influence CNS metastasis over time.

	Univariate		Multivariate	
Variable	OR (95% CI)	*p* value	OR (95% CI)	*p* value
Treatment		**0.016**		
ET + CDKI	1		1	
ET alone	2.13 (1.15–3.94)		2.16 (1.16–4.01)	0.015*
Age		0.780		
≤ 55	1			
> 55	0.91 (0.47–1.77)			
Menopausal status		0.394		
Premenopausal	1			
Postmenopausal	1.29 (0.72–2.32)			
Previous chemotherapy		0.593		
Yes	1			
No	1.21 (0.60–2.46)			
Previous ET				
No	1			
Tamoxifen	1.33 (0.64–2.74)	0.447		
Aromatase inhibitors	1.40 (0.43–4.54)	0.965		
Disease‐free interval				
Newly diagnosed disease	1			
Existing disease				
≤ 24 month	1.05 (0.38–2.93)	0.923		
> 24 month	1.16 (0.46–2.94)	0.748		
Lung or liver involvement				
No	1		1	
Liver	0.45 (0.15–1.34)	**0.151**	0.43 (0.15–1.28)	0.130
Lung	1.22 (0.63–2.36)	0.557	1.21 (0.62–2.34)	0.583
Both	2.04 (0.54–7.75)	0.297	1.94 (0.50–7.49)	0.336
Bone involvement				
No	1			
Any	0.79 (0.39–1.57)	0.495		
Only	1.17 (0.56–2.46)	0.671		
First on‐study ET		0.996		
Aromatase inhibitors	1			
Fulvestrant	1.00 (0.52–1.92)			

*Note*: OR represents the odds ratio for the incidence of CNS metastasis over time.

Abbreviations: CDKI, cyclin‐dependent kinase 4/6 inhibitor; CI, confidence interval; CNS, central nervous system; ET, endocrine therapy; OR, odds ratio.

Bold values indicate p < 0.2, which were included in the subsequent multivariate analysis.

*p <0.05

In total, progression events occurred in 46.5% of patients (100 of 215 patients) and 81.8% of patients (180 of 220 patients) in the ET + CDKI and ET alone groups, respectively. A total of 16.7% of deaths (36 of 215 patients) occurred in the ET + CDKI group, and 42.7% of deaths (94 of 220 patients) in the ET alone group. Median PFS was longer for ET + CDKIs compared with ET alone (23 months [95% CI, 15.3–30.7] vs. 12 months [95% CI, 10.9–13.1], respectively; HR, 0.40; 95% CI, 0.32–0.51; Figure 1B). The median OS time was 67 months (95% CI, 63.3–70.4) for ET + CDKIs, while it was 44 months (95% CI, 41.9–46.1) for ET alone (HR, 0.52; 95% CI, 0.30–0.60; Figure 1C).

**FIGURE 2:**
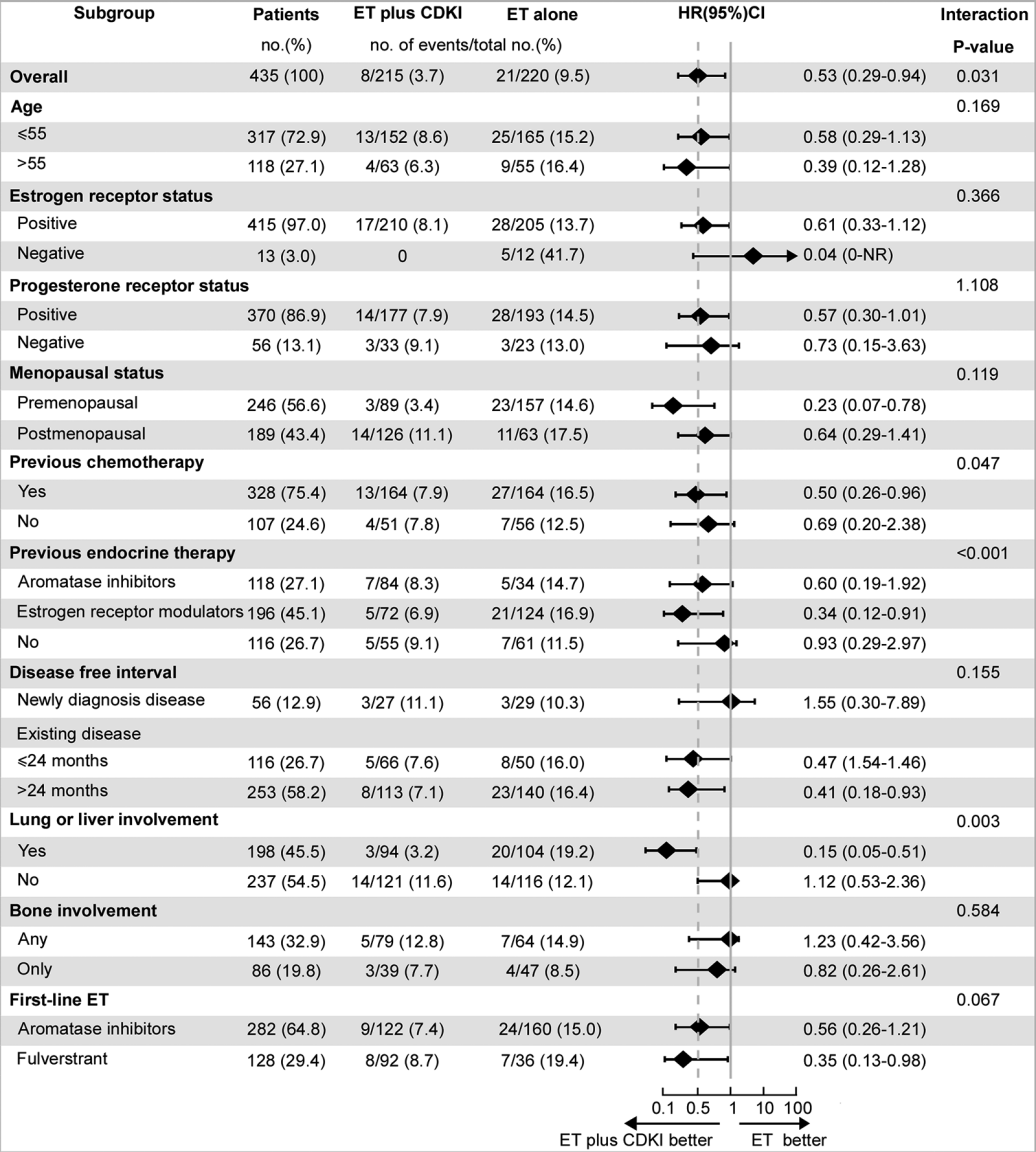
Subgroup analysis of CNSM‐FS.

An exploratory subgroup analysis was carried out (Figure 2). Compared with ET alone, ET + CDKIs extend the CNSM‐FS period across all subgroups. There was a significant interaction effect between previous chemotherapy, previous ET, lung or liver involvement, and the treatment modality (ET + CDKI vs. ET) upon CNSM‐FS. Patients with (neo)adjuvant chemotherapy, adjuvant Tamoxifen, and lung or liver involvement gain more CNS metastasis‐free benefit than others. However, these results need to be interpreted with caution, given the small sample sizes in some subgroups.

## 3 Discussion

To our knowledge, this represents the first multicenter, retrospective cohort study specifically evaluating the potential of CDKIs for CNS metastasis prevention in ABC. Our findings demonstrate that first‐line ET combined with CDKIs significantly reduces CNS metastasis incidence compared to ET alone. These results suggest a previously underrecognized neuroprotective effect of CDKIs that could substantially impact clinical practice. These observations underscore the necessity for additional validation through randomized controlled trials to establish causal relationships and optimize therapeutic strategies for CNS metastasis prevention.

The incidence of CNS as the first metastasis site was 3.7% in the ET + CDKI group and 9.5% in the ET alone group, which directly confirmed the excellent effect of CDKIs on preventing CNS metastasis. Notably, most of the previous literature commonly reported on CNS progression at any time rather than as the first site of progression [12, 15, 16]. In our study, the incidence of CNS progression at any time in the ET + CDKI group (7.9%) was lower than anticipated; however, the incidence of CNS metastasis in the ET group (15.5%) matched that reported in previous studies (approximately 15%) [15]. It is important to note that in this study, all patients were free of BM at baseline, as determined by enhanced brain CT or MRI scans at the initiation of treatment; the other baseline characteristics were well‐balanced. Therefore, the lower incidence of metastasis to the CNS over time can be attributed to the treatment with CDKIs. However, caution should be exercised while making this conclusion and extending it to other studies because our study is based on a very small number of patients with CNS metastases.

CDKs, along with their protein regulator cyclin D1 (encoded by CCND1)—a gene directly regulated by ER signaling—play a key role in controlling cell‐cycle progression. The proposed mechanisms include the inhibition of CDK4 and CDK6, blocking Rb phosphorylation, and preventing tumor cells from entering the S phase, thereby inhibiting tumor growth [26]. Overexpression of CDK 4/6 and amplification of CCND1 are commonly observed in HR‐positive BCs, where they serve as critical drivers of endocrine resistance [27, 28]. The antimetastatic effect of CDKIs can be explained by several key findings. Firstly, alterations in the CDK pathway are potential factors that promote the spread of tumor cells to the CNS. This is supported by a recent genomic analysis of patient‐matched primary tumors and BM, which identified the CDK pathway as a potential contributor to CNS dissemination across tumors of diverse histologies [29, 30]. Moreover, enzymes in the CDK pathway have been recognized as both oncogenic drivers and therapeutic targets in several cancer types [31]. Collectively, these findings indicate that CDK pathway alterations could serve as a promising therapeutic target for BM. Secondly, CDKI treatment prevents CNS metastasis, possibly, in part, due to its ability to continually suppress HR+ tumors, which can delay the development of CNS metastases by controlling extracranial disease. Additionally, multiple preclinical and clinical studies [18, 19, 20, 21, 22] have indicated that CDKIs can penetrate the BBB; that is, they can directly affect tumor cells within the brain. For example, abemaciclib cannot traverse out of the BBB via P‐gp and BCRP efflux transporters, significantly extending survival in an orthotopic U87MG glioblastoma xenograft model [20, 23]. Clinical research results indicate that potential therapeutic doses of ribociclib can be achieved in both cerebrospinal fluid and tumor tissue [24]. This suggests that CDK4/6 inhibitors can also provide clinical benefits for HR+/HER2− BC patients with BM.

We also evaluated the efficacy of ET + CDKI versus ET in patients with ABC. The observed median PFS durations for the ET + CDKI and ET groups were 23 and 12 months, respectively. Similar to a previous study [31], our study also reported that ET + CDKIs were associated with significantly improved OS compared with that achieved using ET alone. Furthermore, our study showed that ET + CDKIs provided a significantly longer PFS period and tended to afford a longer OS time compared to that obtained with ET alone.

The current analysis has certain limitations as well. First, it is a retrospective and exploratory evaluation of CNS metastases; the results need to be carefully interpreted, given the retrospective and non‐randomized nature of this study. Second, the sample size was relatively small. Additionally, the absence of any mandatory or prespecified brain imaging requirement may have underestimated the incidence of CNS metastasis in both treatment groups. Furthermore, the proportion of patients diagnosed with CNS metastasis is significantly influenced by the length of the follow‐up period. This may introduce bias among patients who are alive at the time of the present study, as these individuals might be diagnosed with CNS metastasis either currently or in the future. Moreover, a potential bias may favor a lower incidence of CNS progression in the ET alone group, as this population had a shorter OS, reducing the likelihood of detecting CNS progression that could have occurred following the first site of progression. Importantly, since the study excluded patients with progressive CNS metastases, it was unable to evaluate whether CDKIs can induce CNS tumors, reduce CNS tumor size, or halt disease progression in the presence of active CNS disease. Despite these limitations, this is the only reported study concerning CDKIs’ CNS metastasis prevention effect in patients with ABC receiving ET.

In conclusion, our analysis suggests that ET + CDKIs can reduce the risk of CNS progression, which provides valuable insights for clinicians, aiding them in considering the potential benefits of combining ET with CDKIs when selecting treatment regimens, particularly for patients at high risk of CNS metastases. Furthermore, it is crucial to acknowledge the limitations of our study, including its retrospective and non‐randomized nature and the relatively small sample size mentioned above. Our study underscores the necessity for future research, both prospective and retrospective, to definitively establish the observed effects of CDK4/6 inhibitors on CNS metastasis risk and to explore the underlying mechanisms. Larger, prospective studies are essential. These studies would not only validate our findings but also provide a more comprehensive understanding of how CDKIs operate in HR+/HER2− ABC. We urge to prioritize and conduct such prospective research to advance our knowledge and improve clinical outcomes for patients at risk of CNS metastases.

## 4 Methods

### 4.1 Study Design and Patients

We performed the retrospective study in three tertiary hospitals, that is, Sun Yat‐sen University Cancer Center, Guangdong Provincial Hospital of Traditional Chinese Medicine, and Sun Yat‐sen Memorial Hospital, Sun Yat‐sen University.

The patients ranged in age from 18 to 70 years for cases where histology or cytology had verified HR+/HER2− BC, that is, either recurrent or metastatic. Patients were not included if they had a history of receiving a CDKI, systemic chemotherapy, or ET for advanced disease. Additionally, those who had undergone neoadjuvant or adjuvant ET with a disease‐free interval (DFI) of less than 8 months were not considered. Patients with CNS metastasis at baseline were also excluded.

### 4.2 Procedures

In order to confirm ABC, all patients included in our study were evaluated carefully before the study by performing a medical history assessment, ultrasonic imaging, computed tomography (CT), or magnetic resonance imaging (MRI) scan. Pathological slides from metastatic tissue samples were reviewed to confirm HER2 and HR statuses. If metastatic tissue samples were unavailable, slides from the most recent early BC sampling were used. The review was conducted by three experienced pathologists. CDKI was administered orally with the following dose: ribociclib (600 mg per day, following a regimen of 3 weeks on and 1 week off within a 28‐day treatment cycle), palbociclib (100 or 125 mg per day for 3 weeks, followed by 1 week off), and abemaciclib (100 or 150 mg twice daily on a continuous dosing schedule). ET included aromatase inhibitors (letrozole at a dose of 2.5 mg, anastrozole at a dose of 1 mg, or exemestane at a dose of 25 mg), estrogen receptor modulators (ERM, tamoxifen at a dose of 20 mg or toremifene at a dose of 60 mg), and fulvestrant (500 mg intramuscularly every 14 days for the first three injections and then every 28 days).

Baseline characteristics (e.g., age at diagnosis, menopausal status), HR and HER2 status, Ki‐67 scores, sites of metastases, surgery time, prior treatment, time of first relapse and progression, image data, first‐line therapy, treatment arm (ET + CDKIs or ET alone), subsequent treatments, survival status, and the last follow‐up time were collected from each center's electronic medical records system. The occurrence of CNS metastases during follow‐up was documented through a medical chart review. CNS metastasis was detected following the onset of neurologic symptoms and was confirmed through brain imaging, primarily using MRI or, when unavailable, CT scans.

### 4.3 End Points

The primary end point was to evaluate the incidence of CNS as the first metastasis site (defined as the occurrence of CNS metastasis when the ET ± CDKI treatment progresses), CNSM‐FS (the interval between the diagnosis of ABC and the diagnosis of CNS metastasis), and the incidence of CNS metastasis over time (defined as the occurrence of CNS metastasis at any time after ET ± CDKI treatment). The secondary end points included PFS (the time when ET ± CDKIs were administered the first time to the date of disease progression or death from any cause) and OS (the time when ET ± CDKIs were administered the first time to the date of disease progression or death from any cause).

### 4.4 Statistical Analysis

Categorical variables were presented as frequencies and percentages, and continuous variables were expressed as median and interquartile range (IQR). Baseline characteristics were compared between the two treatment arms using appropriate statistical tests. For continuous variables, between‐group differences were assessed using the nonparametric Wilcoxon rank‐sum test (Mann–Whitney *U* test), which is more appropriate for non‐normally distributed data. Categorical variable comparisons were performed using Pearson's *χ* ^2^ test. In cases where the expected cell count was less than 5, Fisher's exact test was employed as an alternative to maintain statistical validity.

The primary outcomes of interest, namely the incidence of CNS metastasis as the first metastatic site and the incidence of CNS metastasis over time, were evaluated as proportions in each treatment group. Pearson's chi‐square test was used to assess the differences between the two groups, thereby determining the treatment‐related effects. Univariate logistic regression models were constructed to examine the associations between individual variables and the incidence of CNS metastasis as the first metastatic site and over time. Subsequently, multivariable logistic regression models were developed to adjust for potential confounding factors and to assess the independent effect of adjuvant therapy on the outcomes of interest. Variables with a *p*‐value less than 0.2 in the univariate analysis were considered for inclusion in the multivariable models. For categorical variables with more than two categories, at least one category must have met this criterion to be included as an adjustment factor.

Survival outcomes, including CNSM‐FS, PFS, and OS, were estimated using the Kaplan–Meier method and compared between treatment groups using the log‐rank test. A subgroup analysis was designed to evaluate CNSM‐FS in patients receiving ET in combination with CDKIs versus those on ET alone, focusing on clinically relevant subgroups using univariable Cox regression models. The results for each subgroup were presented using forest plots, including the hazard ratio (HR) with 95% confidence intervals (CI) for the two treatment arms, as well as the *p*‐value (Wald test) indicating the interaction between the subgroup and the treatment.

All reported *p* values were two‐sided, with a predefined significance threshold of *p* < 0.05 indicating statistical significance. All statistical calculations and visualizations were executed by SPSS (version 20.0) and R (version 4.2.2).

## Author Contributions

Z.‐Y.Y. and C.X. accessed and took responsibility for the data. Z.‐Y.Y. and C.X conceptualized the data. C.J., L.W., Y.‐Y.Z., Y.‐Y.H., J.‐L.Z., Q.‐J.C curated the data. Y.‐L.W. and Y.‐Y.Z provided formal analysis and methodology. Y.‐L.W., X.‐W.B., X.‐W.Z. and S.‐F.W. wrote the original draft. Z.‐Y. Y. and X.‐W.Z. received the funding acquisition. Y.‐L.W provided visualization. All authors contributed to the review and editing of the original draft. All authors have read and approved the final manuscript.

## Ethics Statement

Our study protocol was approved by the Ethics Committee of the three aforementioned institutions (Approval No. B2023‐172‐01, 2020‐KY‐063, ZF2023‐374‐01). Due to the retrospective nature of the study, the requirement for written informed consent from patients was waived. All personal data were processed anonymously in accordance with the Helsinki Declaration of 1964 and its subsequent amendments.

## Conflicts of Interest

The authors declare no conflicts of interest.

## Data Availability

The data analyzed in this study is available from the corresponding author, Zhong‐Yu Yuan (E‐mail: yuanzhy@sysucc.org.cn), upon reasonable request.
